# Relationship of CHA_2_DS_2_-VASc and CHADS_2_ Score to Left Atrial Remodeling Detected by Velocity Vector Imaging in Patients with Atrial Fibrillation

**DOI:** 10.1371/journal.pone.0077653

**Published:** 2013-10-17

**Authors:** Yihui Li, Wenyuan Ding, Hua Wang, Nianpeng Song, Leyu Lin, Zhihao Wang, Ming Zhong, Yun Zhang, Wei Zhang

**Affiliations:** 1 Key Laboratory of Cardiovascular Remodeling and Function Research, Chinese Ministry of Education and Chinese Ministry of Health, Jinan, P.R. China; 2 Qilu Hospital of Shandong University, Jinan, P.R. China; Temple University, United States of America

## Abstract

**Background:**

The CHADS_2_/CHA_2_DS_2_-VASc scores are used to predict thrombo-embolic/stroke in patients with nonvalvular atrial fibrillation (AF). Nevertheless, limited data are available regarding the association between these risk stratification for stroke and left atrial (LA) remodeling status of AF patients. The purpose of this study was to explore the association between these scores and LA remodeling status assessed quantificationally by echocardiography in AF patients.

**Methods:**

One hundred AF patients were divided into 3 groups based on the CHA_2_DS_2_-VASc/CHADS_2_ score: the score of 0 (low stroke risk), the score of 1 (moderate stroke risk) and the score of ≥2 (high stroke risk). All patients were performed through conventional and velocity vector imaging echocardiography. Echocardiographic parameters: maximum LA volume index (LAVImax), LA total emptying fraction (LAEFt) and LA mean strain were obtained to assess quantificationally LA remodeling status.

**Results:**

On categorizing with CHA_2_DS_2_-VASc, the score of 1 group showed augment in LAVImax and attenuation in LA mean strain derived from VVI, compared with the score of 0 group (LAVImax: 40.27±21.91 vs. 26.79±7.87, *p*=0.002; LA mean strain: 15.18±6.36 vs. 22±8.54, *p*=0.001). On categorizing with the CHADS_2_ score, similar trends were seen between the score of ≥2 and 1 groups (LAVImax: 43.72±13.77 vs. 31.41±9.50, *p*<0.001; LA mean strain: 11.01±5.31 vs. 18.63±7.00, *p*<0.001). With multivariate logistic regression, LAVImax (odds ratio: 0.92 , 95% C=I: 0.85 to 0.98, *p*= 0.01) and LA mean strain reflecting LA remodeling (odds ratio: 1.10, 95% CI: 1.02 to 1.19, *p*=0.01) were strongly predictive of the CHA_2_DS_2_-VASc score of 0.

**Conclusions:**

The superiority of the CHADS_2_ score may lay in identifying LA remodeling of AF patients with high stroke risk. Whereas, the CHA_2_DS_2_-VASc score was better than the CHADS_2_ score at identifying LA remodeling of AF patients presenting low stroke risk.

## Introduction

Atrial fibrillation (AF) is one of the most common arrhythmias in clinical practice [1]. The incidence of thrombo-embolic/stroke increases by an average fivefold in the presence of AF [2]. To stratify thrombo-embolic risks in patients with nonvalvular AF and to identify patients eligible for anticoagulation, two stroke risk stratification schemes have been widely applied in clinical practice, including the CHADS_2_ score (Congestive heart failure, Hypertension, Age ≥75, Diabetes, Stroke [doubled]) [3] and the CHA_2_DS_2_-VASc score (Congestive heart failure, Hypertension, Age ≥75 [doubled], Diabetes, Stroke [doubled], Vascular disease, Age 65–74, and Sex category [female]) [4]. 

Many studies have demonstrated that the risk factors in the two stratification schemes also contribute to triggering a slow but progressive process of LA structural and functional changes in every AF patients, termed as LA remodeling [5]. Meanwhile, LA remodeling is a critical substrate of thrombo-embolic tendency in AF [6]. Therefore, the CHADS_2_ and CHA_2_DS_2_-VASc score might represent LA remodeling status, and a higher stroke risk possibly imply a more serious atrial remodeling status. Precise assessment of LA remodeling could improve our ability to predict the risk of developing stroke and the response to treatments in patients with this arrhythmia [7]. However, their precise association is incompletely understood. Compared with pure conventional echocardiograph, the combination of velocity vector imaging (VVI) and conventional echocardiograph could offer more comprehensive echocardiographic parameters quantificationally reflecting status of LA remodeling, such as atrial volume, ejection fraction and strain [7–10].

Our study was undertaken to determined the association of the CHADS_2_/CHA_2_DS_2_-VASc score and the LA remodeling status quantificationally evaluated by both conventional echocardiograph and VVI in AF. In addition, we also sought to fully assess incremental value over various echocardiographic parameters for predicting the stroke risk.

## Methods

It’s a single-center, retrospective, cross-sectional study. The study was approved by the Ethics Committee of Qilu Hospital of Shandong University, Jinan, China. All subjects gave written informed consent to participate in this study. One hundred consecutive patients with nonvalvular AF attending Qilu Hospital in Shandong Province between March 2010 and July 2012 were included in this study. All AF patients underwent routine echocardiographic study after initial treatment in the E.R. or in the cardiology department of the Qilu Hospital. Subjects with valve disease, primary myocardial and pericardial diseases were excluded. To stratify the subjects, comorbid conditions and other risk factors such as congestive heart failure, hypertension, age ≥75, diabetes, history of stroke, vascular disease, age 65–74, and sex category were taken into account according to the CHA_2_DS_2_-VASc or CHADS_2_ scheme criteria [5,11]. In both CHA_2_DS_2_-VASc or CHADS_2_ scheme systems, a score of 0 was categorized into low risk, a score of 1 was moderate risk , and score ≥2 was high risk [12].

All subjects were examined in the left lateral decubitus using the Siemens Sequoia c512 ultrasound machine, revision 8.0 (Siemens Medical Systems, Mountain View, CA, USA) and a 4 V1c transthoracic transducer during quiet respiration. All echocardiograms were performed by 2 experienced operators (Yihui Li, Nianpeng Song) with no previous specific information about the study objects. 

All echocardiographic images were recorded by the Digital Imaging and Communication in Medicine (DICOM) and formatted file images were then exported to a personal computer. All analyses were performed off line with software Syngo US Workplace (Siemens Medical Solutions, Mountain View, CA, USA, revision 3.0).

### Conventional echocardiography

Two-D images were acquired from the apical 4-chamber and 2-chamber views according to standard techniques [13]. 

All LA volumes were calculated from the apical 4-chamber and 2-chamber views using the biplane method of discs[14]. All LA volumes were indexed to body surface area for LA volumes index (1). Maximum LA volume index (LAVImax) was defined as the largest LA volume index, in ventricular systole just before mitral valve opening; (2) minimal LA volume (LAVImin) was defined as the smallest LA volume index, after mitral valve closure. The LA emptying parameters were derived from LA volumes index: LA total emptying fraction (LAEFt= (LAVImax—LAVImin) ╱LAVImax) [15].

Left ventricular systolic and diastolic function: LV ejection fraction (LVEF) was calculated using the biplane Simpson’s rule [16]. The left ventricular diastolic function was assessed by E/e’sep, the ratio of mitral inflow E velocity to myocardial e’sep velocity in the septal mitral annulus [17,18].

### Velocity vector imaging (VVI)

**Figure 1:**
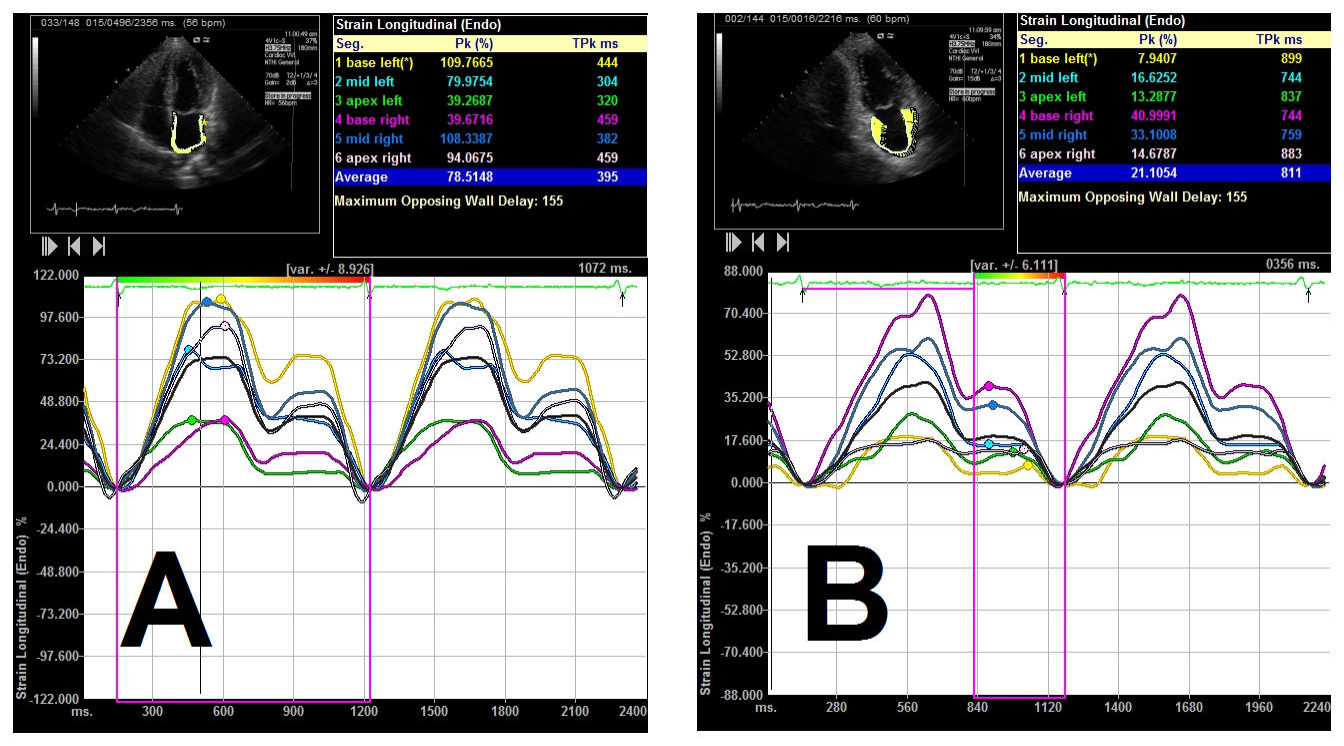
Examples of strain wave of curves of different colors representing the strain of different segments of atrial walls. Imagine A (from 4-chamber view) and B (from 2-chamber view) show the strain wave from a paroxysmal AF subjects.

High quality apical 4-chamber and 2-chamber gray scale views images were obtained on the condition of quiet breath and a stable and distinct ECG recording. The endocardium of the LA walls on apical 2-chamber, 4-chamber view was manually traced starting from the medial to the lateral mitral annulus and was tracked by the VVI software along the border throughout 2 to 3 cardiac cycles. Accuracy of border tracking was manually verified and adjusted if needed. The tracing was performed several times to achieve the most accurate endocardial border. Thus, strain versus time curves were generated from these regions of interest (Figure 1). Strain measurements performed in the myocardium of the 8 LA segments (each basal, mid segments of LA septal, lateral, inferior, anterior wall) were calculated by averaging values for LA mean strain. The apical segments’ data with pulmonary veins and atrial appendage was excluded as recommended [7,19]. 

### Statistical Analysis

Continuous data are presented as mean ±SD. Categorical data are summarized as frequencies and percentages. One-way ANOVA of normal distributional continuous data was used to compare the differences among groups of subjects. Comparison of the prevalence of comorbid conditions (hypertension, coronary artery disease, heart failure, etc.) were made using the χ^2^ test or Fisher exact tests if necessary. Univariate and multivariate linear regressions were used to determine the variables for predicting LA mean strain [20]. Univariate and multivariate logistic regression analyses in AF to determine the predictors of the CHA_2_DS_2_-VASc score of 0. The receiver-operator characteristic (ROC) curve testing diagnostic value of the various echocardiographic parometers model (in which LVEF and E/e’sep reflected the left ventricular function, LAEFt, LAVImax and LA mean strain reflected the left atrial function) was used to predict the CHA_2_DS_2_-VASc score of 0 in AF group.

Bland-Altman plots were used to assess the reproducibility of intraobserver and interobserver. A performer (Yihui Li) repeated VVI and obtained strain data of 10 randomly selected patients at two different time points to determine the intraobserver reproducibility. Another performer (Nianpeng Song) obtained VVI data of the same 10 patients independently for the interobserver reproducibility. The mean bias and limits of agreement (1.96±SD) from Bland-Altman plot are presented for the reproducibility of intraobserver and interobserver. All statistical analyses were performed using SPSS 18.0 software (SPSS Inc., Chicago, Illinois, USA). *p* values <0.05 were considered statistically significant.

## Results

### Study population

Baseline characteristics for the overall study population are presented in Table 1. There was no statistical difference in duration of AF, lab parameters. Compared with the CHA_2_DS_2_-VASc score of 0 score and 1 score group, the CHA_2_DS_2_-VASc score of ≥2 group had significantly increased systolic and diastolic blood pressure (*p*<0.01~0.001), persistent AF (*p*<0.05~0.01), stroke events after AF (*p*<0.05~0.01) and angiotensin ii receptor blockers (ARB) drug utilization (*p*<0.01) .

**Table 1:** Comparison of clinical characteristics in AF with different CHA_2_DS_2_-VASc score.

	**CHA_2_DS_2_-VASc**								
	**0**			**1**			**≥2**		
**Clinical characteristics**	**(n = 25)**			**(n = 27)**			**(n = 48)**		
**Age>75**	**0**	**(0%)**		**0**	**(0%)**		**4**	**(8.3%**)	
**Age 65–74 y**	**0**	**(0%)**		**6**	(22.2%)*		**20**	**(41.7%**)***	
**Congestive heart failure**	**0**	**(0%)**		**1**	**(3.7%)**		**19**	(39.6%)***††	
**Hypertension**	**0**	**(0%)**		**7**	(25.9%)*		**28**	(58.3%)***††	
**Diabetes**	**0**	**(0%)**		**6**	**(22.2%)**		**7**	**(14.6%**)	
**Stroke**	**0**	**(0%)**		**1**	**(3.7%)**		**18**	(37.5%)***††	
**Vascular disease**	**0**	**(0%)**		**1**	**(3.7%)**		**2**	**(4.2%**)	
**Female gender**	**0**	**(0%)**		**6**	(22.2%)*		**25**	(52.1%)***††	
**SBP, mmHg**	**124.8**	**±**	**8.83**	**130.89**	**±**	**19.69**	**144.19**	**±**	16.98***†††
**DBP, mmHg**	**76.2**	**±**	**7.58**	**82.26**	**±**	**13.55**	**84.17**	**±**	13.73**
**Ventricular rate,prm**	**81.63**	**±**	**16.23**	**80.48**	**±**	**24.98**	**80.69**	**±**	**16.65**
**BMI,kg/m^2^**	**25.7**	**±**	**2.71**	**25.25**	**±**	**4.45**	**26.92**	**±**	**4.11**
**Waist-Hip Ratio**	**0.92**	**±**	**0.04**	**0.89**	**±**	**0.06**	**0.91**	**±**	**0.06**
**FBG, mmol/L**	**5.24**	**±**	**0.59**	**6.08**	**±**	**2.67**	**5.57**	**±**	**1.19**
**TC,mmol/L**	**1.66**	**±**	**0.84**	**1.58**	**±**	**1.43**	**1.56**	**±**	**0.85**
**TG,mmol/L**	**4.62**	**±**	**0.72**	**4.74**	**±**	**1.3**	**4.72**	**±**	**1.31**
**HDL,mmol/L**	**1.16**	**±**	**0.27**	**1.25**	**±**	**0.34**	**1.21**	**±**	**0.28**
**LDL,mmol/L**	**2.59**	**±**	**0.5**	**2.62**	**±**	**0.87**	**2.74**	**±**	**0.99**
**AF history, month**	**67.77**	**±**	**69.15**	**47.44**	**±**	**38.8**	**71.68**	**±**	**75.71**
**Persistent AF**	**8**	**(32%)**		**11**	**(40.7%)**		**31**	(64.6%)**†	
**Stroke events after AF**	**0**	**(0%)**		**1**	**(3.7%)**		**13**	(27.1%)**†	
**Previous antiarrhythmic drugs**	**21**	**(84%)**		**19**	**(70.4%)**		**39**	**(81.3%)**	
**Previous antiplatelet drugs**	**11**	**(44%)**		**16**	**(59.3%)**		**33**	**(68.8%)**	
**Previous anticoagulation drugs**	**8**	**(32%)**		**9**	**(33.3%)**		**18**	**(37.5%)**	
**CCB**	**2**	**(8%)**		**6**	**(22.2%)**		**15**	**(31.3%)**	
**ACEI**	**5**	**(20%)**		**4**	**(14.8%)**		**14**	**(29.2%)**	
**ARB**	**2**	**(8%)**		**5**	**(18.5%)**		**18**	(37.5%) **	

SBP, systolic blood pressure; DBP, diastolic blood pressure; BMI, Body mass index; FBG, Free blood glucose; TG, total triglyceride; TC, total cholesterototal; HDL, high-density lipoprotein cholesterol; LDL, low-density lipoprotein cholesterol; ACEI, angiotensin-converting enzyme inhibitor; ARB, angiotensin ii receptor blockers, CCB, calcium channel blocker; *p<0.05 compared with 0 score group; **p<0.01 compared with 0 score group; ***p<0.001 compared with 0 score group; †*p*<0.05 compared with 1 score group;†† *p*<0.01 compared with 1 score ;††† *p*<0.001 compared with 1 score group.

### Left ventricular systolic and diastolic function

Conventional left ventricular functional parameters from the study population are presented in Table 2. AF with CHA_2_DS_2_-VASc score ≥2 had lower LVEF and higher E/e’sep significantly (p<0.01). There were no significant differences between the CHA_2_DS_2_-VASc score of 0 and CHA_2_DS_2_-VASc score of 1 group about left ventricular function.

**Table 2:** Left ventricular systolic and diastolic function of AF with different CHA_2_DS_2_-VASc score.

	**CHA_2_DS_2_-VASc score**								
	**0**			**1**			**≥2**		
**Left ventricular function**	**(n=25)**			**(n=27)**			**(n=48)**		
**LVEF,%**	**61**	**±**	**7**	**58**	**±**	**8**	**54**	**±**	1**
**E/A**	**1.12**	**±**	**0.41**	**1.02**	**±**	**0.33**	**1.04**	**±**	**0.46**
**E/e`(SEP)**	**5.12**	**±**	**2.17**	**6.01**	**±**	**1.73**	**6.96**	**±**	2.41**

LVEF, LV ejection fraction; E/A, the ratio of Early (E) and late (A) wave velocities from Pulsed Doppler mitral inflow; E/e`(sep), the ratio of mitral inflow E velocity to myocardial e`sep velocity in the mitral annulus; **p<0.01 compared with 0 score group.

### Left atrial remodeling status categorized by CHADS_2_ score

**Figure 2:**
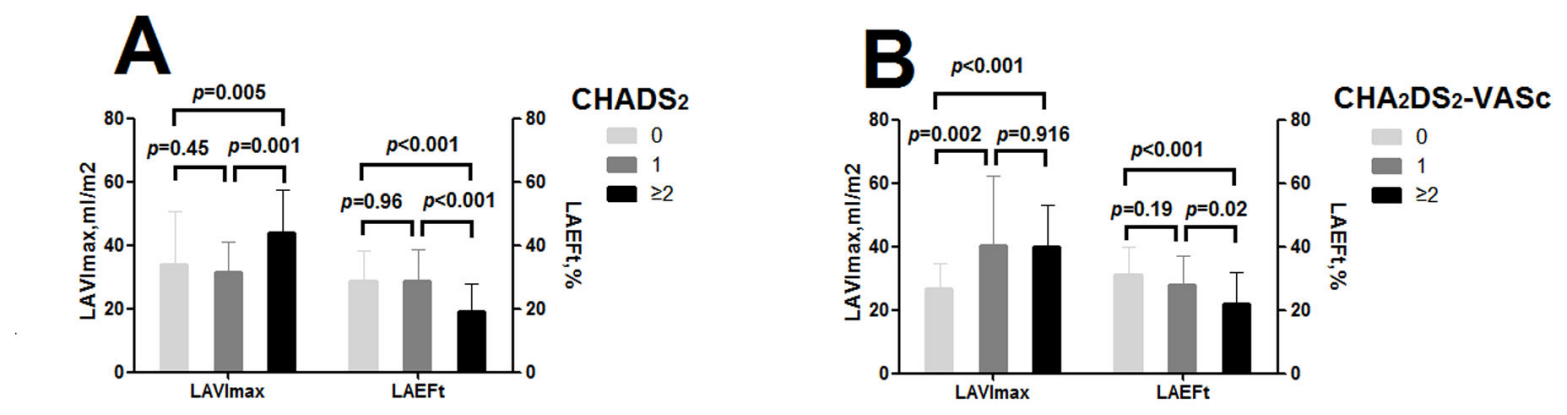
Left atrial volume index and ejection fraction of AF with different stroke risk score. Low(CHADS_2_/CHA_2_DS_2_-VASc score of 0), moderate (CHADS_2_ /CHA_2_DS_2_-VASc score of 1), and high (CHADS_2_/CHA_2_DS_2_-VASc score of ≥2) risk; Maximum LA volume index; LAEFt , LA total emptying fraction; LAEFp.

**Figure 3:**
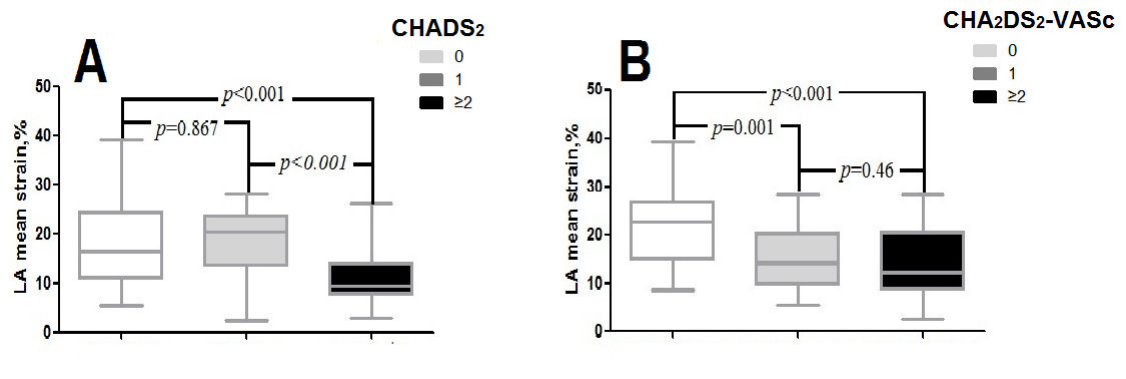
Distribution of LA mean strain of AF with different stroke risk score. Low (CHADS_2_/CHA_2_DS_2_-VASc score of 0), moderate (CHADS_2_ /CHA_2_DS_2_-VASc score of 1), and high (CHADS_2_/CHA_2_DS_2_-VASc score of ≥2) risk; The central box represents the values from the lower to upper quartile (25th to 75th percentile). The middle line represents the median. The whiskers extend from the minimum to the maximum value.

On categorizing AF patients based on the CHADS_2_ score, the difference of LAVImax and LAEFt between 1 score and 0 score group wasn’t significant (LAVImax: 31.41±9.50 vs. 33.98±16.70, *p*=0.45; LAEFt: 28.83±9.90 vs. 28.71±9.62, *p*=0.96) (Figure 2.A). Similarly, no significant difference of LA mean strain between the CHADS_2_ score of 1 group and 0 group could be shown (18.63±7.00 vs. 18.33±8.57, *p*=0.867) (Figure 3.A).

Whereas LAVImax was increased and LAEFt was reduced significantly in ≥2 score group compared with 1 score group (LAVImax: 43.72±13.77 vs. 31.41±9.50, *p*<0.001; LAEFt: 19.18±8.53 vs. 28.83±9.90, *p*<0.001) (Figure 2.A). And significant decrease in LA mean strain was present in ≥2 score group compared with 1 score group. (11.01±5.31 vs. 18.63±7.00, *p*<0.001 ) (Figure 3.A).

### Left atrial remodeling status categorized by CHA_2_DS_2_-VASc score

Categorized by CHA_2_DS_2_-VASc, the 1 score group was significantly higher in LAVImax (40.27±21.91 vs. 26.79±7.87, *p* =0.002) compared with 0 score group)(Figure 2.B). Likewise, LA mean strain in the 1 score group decreased significantly compared with 0 score group (15.18±6.36% vs. 22±8.54%, *p*=0.001)(Figure 3.B). LAEFt was lower in 1 score group compared with 0 score group (27.51±9.14 vs. 31.08±9.45, *p*=0.19), but the difference didn’t reach statistical significance (Figure 2.B).

Nevertheless, there were no significant difference of left atrial echocardiographic values between 1 score and ≥2 score group (LAVImax: 40.27±21.91 vs. 39.80±13.35, *p*=0.916; LAEFt: 27.51±9.14 vs. 22.19±10.05, *p*=0.02; LA mean strain:15.18±6.36% vs. 13.86±7.10%, *p*=0.46 ) (Figure 2.B, 3.B).

### The correlation of echocardiographic parameters assessing left atrial remodeling

Univariate and multivariate regression analysis was used to determine the predictors of LA mean strain. The results of the regression analysis are shown in Table 3. Multivariate analysis revealed LA mean strain was correlated with LAVImax and LAEFt (R^2^ =0.559, *p* =0.03 and *p*<0.001 for LAVImax and LAEFt, respectively).

**Table 3:** Univariate and multivariate regression analysis for LA mean strain in AF.

	**Univariate regression**			**Multivariate regression**			
**Variable**	**Coefficient**	*p*		**Coefficient**	**SE**	*p*	**Adjusted R^2^**
**LAVImax**	**-0.17**	**0.13**		**-0.22**	**0.06**	**0.03**	**0.559**
**LAEFt**	**0.47**	**0.02**		**0.63**	**7.77**	**<0.001**	**-**
**LVEF**	**0.07**	**0.61**		**-**	**-**	**-**	**-**
**E/e`sep**	**-0.09**	**0.41**		**-**	**-**	**-**	**-**
**Age**	**-0.08**	**0.49**					
**BMI**	**0.11**	**0.33**		**-**	**-**	**-**	**-**
**SBP**	**-0.12**	**0.36**		**-**	**-**	**-**	**-**
**DBP**	**-0.07**	**0.64**		**-**	**-**	**-**	**-**
**Ventricular rate**	**-0.03**	**0.79**		**-**	**-**	**-**	**-**
**Type of AF**	**0.15**	**0.45**		**-**	**-**	**-**	**-**
**History of AF**	**-0.06**	**0.55**		**-**	**-**	**-**	**-**
**FBG**	**-0.06**	**0.55**		**-**	**-**	**-**	**-**

Maximum LA volume index; LAEFt, LA total emptying fraction; LAEFp; LVEF, left ventricular ejection fraction; E/e`(sep), the ratio of mitral inflow E velocity to myocardial e`sep velocity in the mitral annulus; SBP, systolic blood pressure; DBP, diastolic blood pressure; BMI, Body mass index; FBG, Free blood glucose.

### The value over various echocardiographic parameters for predicting the CHA_2_DS_2_-VASc score of 0

Logistic regression analysis in AF identified LAVImax (odds ratio: 0.92 , 95% CI: 0.85 to 0.98, *p*= 0.01) and LA mean strain (odds ratio: 1.10, 95% CI: 1.02 to 1.19, *p*=0.01) as the echocardiographic variables that were associated with greater predictive odds of the CHA_2_DS_2_-VASc score of 0 (Table 4).

**Table 4:** Univariate and multivariate Logistic Regression Analysis for Prediction CHA_2_DS_2_-VASc score=0.

	**Univariate**				**Multivariate**		
**Variable**	**OR**	**95%CI**	*p*		**OR**	**95%CI**	*p*
**LAVImax**	**0.912**	**0.85 - 0.98**	**0.02**		**0.92**	**0.85-0.98**	**0.01**
**LA mean strain**	**1.117**	**1.01 - 1.23**	**0.03**		**1.10**	**1.02-1.19**	**0.01**
**LVEF**	**2.572**	**0 - 24267.71**	**0.84**		**-**	**-**	**-**
**E/e`sep**	**1.016**	**0.81 -1.27**	**0.89**		**-**	**-**	**-**
**LAEFt**	**0.100**	**0 -176.96**	**0.55**		**-**	**-**	**-**

LAVImax, maximum LA volume index; LAEFt, LA total emptying fraction; LAEFp; LVEF, left ventricular ejection fraction; E/e`(sep), the ratio of mitral inflow E velocity to myocardial e`sep velocity in the mitral annulus.

**Figure 4:**
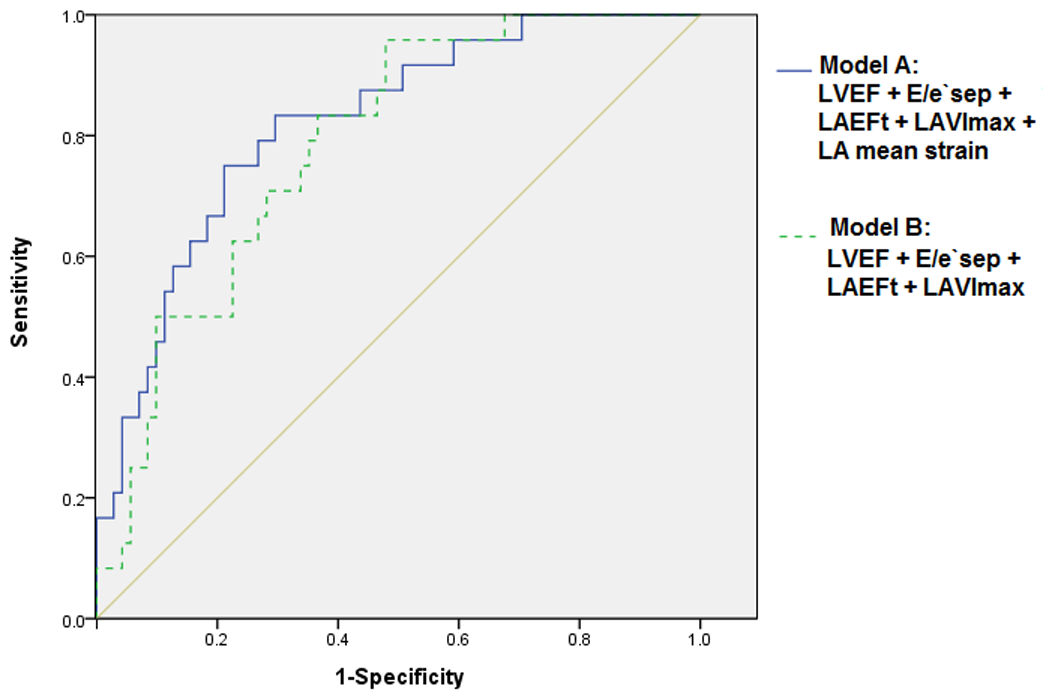
Diagnostic value ROC curve of various cic parameters. The receiver-operator characteristic (ROC) curve testing diagnostic value of left ventricular ejection fraction (LVEF), the ratio of mitral inflow E velocity to myocardial e`sep velocity in the mitral annulus(E/e`sep), Maximum LA volume index(LAVImax), LA total emptying fraction(LAEFt), LA mean strain in predicting CHA_2_DS_2_-VASc score=0 in AF. Two models have different patient numbers that might lead to a small difference in area under the receiver-operator characteristic curve (AUC).Model A: AUC: 0.82, 95%CI: 0.73 to 0.91; Model B: AUC: 0.79, 95%CI: 0.69 to 0.88, *p* =0.59.

The receiver-operator characteristic (ROC) curve was used to test diagnostic value of various echocardiographic parameters models in predicting AF of CHA_2_DS_2_-VASc score of 0. The model A (LVEF + E/e’sep + LAEFt + LAVImax + LA mean strain) increased in the area under the receiver-operator characteristic curve (AUC) about 0.03 from the model B (LVEF + E/e’sep + LAEFt + LAVImax). Unfortunately, the difference didn’t arrive at the statistical significance (the model A: AUC: 0.82, 95%CI: 0.73 to 0.91, the model B: AUC: 0.79, 95%CI: 0.69 to 0.88, *p* =0.59)(Figure 4).

### Intraobserver and interobserver reproducibility

**Figure 5:**
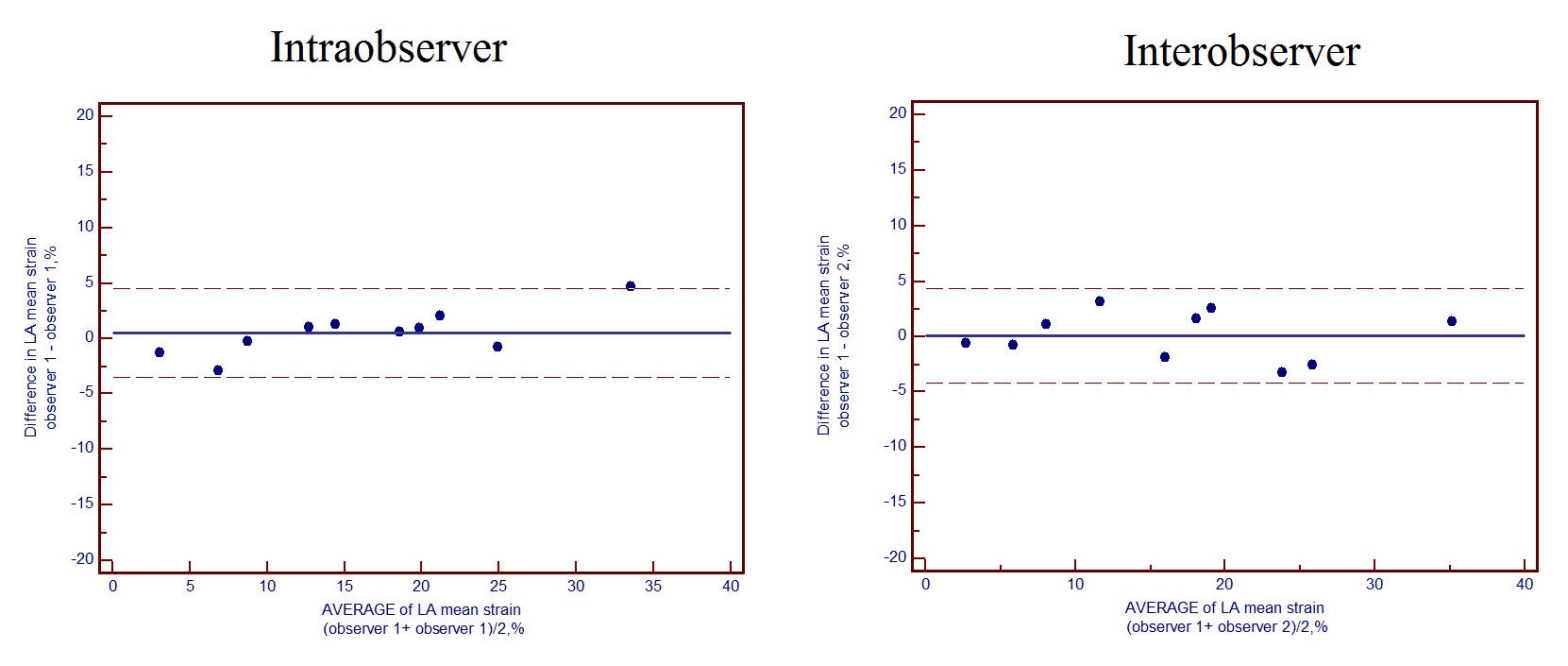
Intraobserver and Interobserver Reproducibility for VVI derived LA Mean strain and LA Mean strain Rate. The mean bias intraobserver was 0.6 (limits of agreement, -3.4 to 4.6,) for LA mean strain. The mean bias interobserver was 0.1 (limits of agreement, -4.2 to 4.4,) for LA mean strain.

Intraobserver and interobserver reproducibility for LA mean strain derived from VVI are presented in Figure 5. The mean bias of intraobserver was 0.6 (limits of agreement, -3.4 to 4.6) for LA mean strain. The mean bias of interobserver was 0.1 (limits of agreement, -4.2 to 4.4) for LA mean strain (Figure 5).

## Discussion

In this study, we demonstrated the relationship between the LA remodeling and CHADS_2_ /CHA_2_DS_2_-VASc score. Both scoring systems may identify LA remodeling status and each has its own merits. The CHA_2_DS_2_-VASc score demonstrated the superiority of distinguishing LA remodeling of AF patients with the low stroke risk from moderate stroke risk. Meanwhile, the CHADS_2_ score had its potential in identifying LA remodeling of AF patients with the high stroke risk. Furthermore, echocardiographic parameters reflecting LA remodeling: LAVImax and LA mean strain derived from VVI showed incremental value in assessing CHA_2_DS_2_-VASc score of 0.

AF and concomitant stroke risk factors have caused extensive and severe abnormalities in ultrastructure, including progressive endocardial denudation, imbalance in collagen synthesis and degradation, and oedematous or fibroelastic infiltration of the extracellular matrix [21]. The ultrastructural changes may enhance atrial dilatation, loss of atrial contractile force and decreased atrial wall compliance [22]. These atrial remodeling in AF patients could lead to further echocardiography changes. 

Traditional approaches assessing atrial remodeling is conventional echocardiography in clinical practice [8,23]. A widely used parameter of assessing LA remodeling by echocardiography is LA Anterior-Posterior diameter in the parasternal long-axis view [7]. However, previous studies have shown little relationship between left atrial diameter by conventional echocardiography and stroke risk in AF [24]. Our results revealed the incremental relationship among LAVImax, LA mean strain and the CHADS_2_ /CHA_2_DS_2_-VASc score. AF patients’ predominant enlargement of LA in the superior-inferior and medial-lateral dimensions could alter LA geometry such that the Anterior-Posterior diameter may not be representative of LA size. There are either good agreement or a tendency for biplane method to underestimate comparative LA volumes [25]. For these reasons, cumulative changes in LA remodeling should be measured by LA volume index in research [26]. LA ejection fraction has been associated with left ventricular systolic and diastolic function [25]. In our study, some AF with CHA_2_DS_2_-VASc score ≥2 had abnormal left ventricular function. So LAEFt couldn’t assess the LA remodeling of AF with left ventricular dysfunction exactly, compared with LA volume index and LA mean strain.

LA strain derived from VVI can reflect dynamic changes that may precede volumetric changes [10]. Because of the combination of speckle tracking (a series of unique B-mode pixel tracking algorithms), mitral annulus motion and tissue-blood border detection, VVI show more feasible and reproducible in assessing LA remodeling status of very thin atrial wall, compared with other echocardiographic methods [7], recently been applied to the evaluation left atrial mechanical dysfunction in coronary artery disease patients [8], regional myocardial dysfunction in patients with acute myocardial infarction [27], have emerged as a quantitative technique for the estimation of myocardial structure and function. Especially, LA strain derived from the VVI is inversely related to LA wall fibrosis, a key hallmark of LA remodeling. Furthermore, the strain wasn’t influenced by age, sex, severity of mitral regurgitation. In our study, LA mean strain was correlated with LA functional parameters (LAVImax and LAEFt) rather than ventricular parameters or clinical characteristics (Table 3). 

The most common stroke risk factors (like the aging, hypertension, heart failure and so on) are most critical upstream concomitant conditions promoting atrial remodeling. Upstream therapy for these coexisting comorbidities can delay, even reverse LA remodeling [5,22]. AF patients with stroke risk factors have more severe abnormalities in ultrastructure [21]. Given all that, more stroke risk factors imply more serious left remodeling status, vice versa. In our study, those AF patients with more stroke risk factors or higher stroke scores had more significant enlargement LA, loss of atrial contraction and reduced strain. 

It is recommended to use several, rather than single doppler echocardiographic technique for the accurate assessment of atria or concomitants events [28,29]. So our study attempted to combine multiple echocardiographic parameters model to predict the CHA_2_DS_2_-VASc score of 0. We also examined the diagnostic value of conventional echocardiographic parameters model after adding LA mean strain, the ROC was 0.82. Disappointedly, the difference didn’t reach the statistical significance (*p* =0.59)(Figure 4). So further studies are necessary to investigate the superiority of strain in predicting the CHA_2_DS_2_-VASc score of 0 and clarify the detailed mechanisms.

North American and European guidelines on atrial fibrillation (AF) are conflicting regarding the classification of patients at low/intermediate risk of stroke [30]. A previous study demonstrated the relationship between LA strain and the stroke risk as quantified with CHADS_2_ score [10], its categorization was based on that CHADS_2_ score < 2 was low, CHADS_2_ score 2 or 3 was moderate, and CHADS_2_ score > 3 was high risk. However, according to the guideline of the 2012 edition of the American College of Chest Physicians (ACCP) Guidelines on Antithrombotic Therapy and Prevention of Thrombosis [31] and the 2010 Guidelines for the management of atrial fibrillation of the European Society of Cardiology (ESC) [5], the CHADS_2_ score of 0 is as low, CHADS_2_ score of 1 is as moderate, and CHADS_2_ score of > 2 is as high risk. Regarding of that, our results revealed that AF with CHADS_2_ score of ≥2 (high stroke risk) showed high risk remodeling additionally. 

The 2012 focused update of the ESC Guidelines for the management of atrial fibrillation strongly recommends a practice shift towards greater focus on identification of ‘truly low-risk’ patients with AF. What’s more, lots of evidence showed that the CHA_2_DS_2_-VASc was better at identifying ‘truly low risk’ patients with AF who wouldn’t develop stroke and thrombo-embolism and a CHADS_2_ score of 0 does not reliably identify AF patients who are ‘truly low-risk’ [11]. Our study revealed that AF with CHA_2_DS_2_-VASc score of 0 might have truly low level LA remodeling, but AF with CHADS_2_ score of 0 may have similar LA remodeling level with CHADS_2_ score of 1, who would subsequently suffer from strokes. Our results could be one reason why real-world cohort data confirmed that not all of the patients with CHADS_2_ score of 0 were low risk, who faced stroke inevitably [12]. 

### Limitations

The population of our study was relatively small. Variations in drug utilization may have resulted in data variability. ARB can prevent and suppress the promotion of atrial structural and electrical remodeling [32,33]. CHA2DS2-VASc score of ≥2 group using more prevalent ARB failed to present better amelioration, so we consider that variations in ARB or other related drugs utilization may not affect atrial remodeling outcomes in our study prominently. Further confirmation is needed in larger, prospective investigations. The bias of echocardiographic parameters in intraobserver and interobserver depends on many factors. Two experienced performers (Yihui Li and Nianpeng Song) obtained and analyzised the echocardiographic data according with standard method. Bland-Altman plot also demonstrated feasible and reproducible results by VVI. The duration from last atrial fibrillation attack may affect the atrial and ventricular function in the paroxysmal AF. Therefore, we performed the echocardiograph to decrease this factor’s influence when the paroxysmal AF patients maintained sinus rhythm after treatments. 

## Conclusions

The superiority of the CHADS_2_ score may lay in identifying LA remodeling of AF patients with high stroke risk. Whereas, the CHA_2_DS_2_-VASc score was better than the CHADS_2_ score at identifying LA remodeling of AF patients presenting low stroke risk. The superiority of the CHA_2_DS_2_-VASc score over CHADS_2_ in indentifying LA remodeling of low stroke risk AF patients may partly account for the fact that the CHA2DS2-VASc is better at identifying ‘truly low stroke risk’ AF patients in real-world cohort.
