# Correction: Relationship of CHA_2_DS_2_-VASc and CHADS_2_ Score to Left Atrial Remodeling Detected by Velocity Vector Imaging in Patients with Atrial Fibrillation

**DOI:** 10.1371/annotation/fb95b7ed-f03b-498b-adda-2683a1b904b7

**Published:** 2013-12-20

**Authors:** Yihui Li, Wenyuan Ding, Hua Wang, Nianpeng Song, Leyu Lin, Zhihao Wang, Ming Zhong, Yun Zhang, Wei Zhang

Affiliation number 1 for all authors is incorrect. The correct affiliation is: Key Laboratory of Cardiovascular Remodeling and Function Research, Chinese Ministry of Education and Chinese Ministry of Health, Department of Cardiology, Qilu Hospital of Shandong University Jinan, P.R. China. 

